# The use of bacterial DNA from saliva for the detection of GAS pharyngitis

**DOI:** 10.1080/20002297.2020.1771065

**Published:** 2020-05-27

**Authors:** Saar Hashavya, Naama Pines, Ayelet Gayego, Avi Schechter, Itai Gross, Alon Moses

**Affiliations:** a Department of Pediatric Emergency Medicine, Hadassah Medical Center, Jerusalem, Israel; b Department of Pediatrics, Hadassah and Hebrew University Hospital, Jerusalem, Israel; c Department of Microbiology and Infectious Diseases, Hadassah and Hebrew University Hospital, Jerusalem, Israel; d Meuhedet Health Maintenance Organization, Israel

**Keywords:** Streptococcus group A, pharyngitis, tonsillitis, throat swab, PCR, saliva

## Abstract

**Background:**

Acute tonsillitis is a very common medical condition. Despite different methods of detection, all tests are based on GAS sampling using a throat swab. However, obtaining the swab can elicit vomiting and is often accompanied by fear and apprehension in children. The aim of this study was to find a non-invasive method for the detection of GAS pharyngitis.

**Methods:**

A classic throat swab was obtained for culture, and a saliva sample was taken from 100 subjects recruited from Meuhedet Health Care Organization clinic. Real time PCR was performed to detect GAS dnaseB specific gene in the saliva samples.

**Results:**

56% of the throat cultures and 48% of the PCR samples were positive for GAS. The overall sensitivity and specificity of the saliva PCR method was 79% and 91% respectively; NPV and PPV were 77% and 92% respectively. When excluding patients who presented on the first day of fever, sensitivity and specificity increased to 90% and 100% respectively. No other anamnestic or clinical findings increased the yield of the test.

**Conclusion:**

Saliva-based PCR amplification of GAS DNA method is effective in detection of GAS pharyngitis. Further studies are needed to achieve detection rates to replace the traditional throat swab-based approach.

## Introduction

Acute tonsillitis is one of the most common medical conditions for which children and young adults seek medical advice. Although most episodes of tonsillitis are caused by viruses, Group A streptococcus (GAS) infection accounts for approximately 15% to 30% of all cases in children and 5% to 15% of all cases in adults [1,2]. The overlap in signs and symptoms of GAS and non-streptococcal tonsillitis makes accurate diagnosis based on clinical assessment alone very challenging even for the astute physician. Accurate diagnosis of streptococcal tonsillitis followed by appropriate antimicrobial therapy is crucial for the prevention of acute rheumatic fever as well as suppurative and other systemic complications [3]. A rapid and correct diagnosis is also important to avoid unnecessary antimicrobial therapy.

Laboratory diagnosis of GAS infections relies on culturing the bacteria from clinical specimens [1].Other methods of GAS detection include the Rapid Antigen Test (RADT), an agglutination or immunoassay test for direct detection of the group A-specific carbohydrate antigen. The specificity of rapid antigen tests is generally high (approximately 95%), but the sensitivity ranges from 70% to 90% [4,5].

Recently, polymerase chain reaction (PCR) amplification-based tests were introduced to identify specific Ribosomal Ribonucleic acid (rRNA) and Deoxyribonucleic Acid (DNA) sequences of *S. pyogenes* in pharyngeal specimens by a single-stranded chemiluminescent nucleic acid probe [2]. A multicenter evaluation study reported a sensitivity and specificity of 99% and 99.6% respectively [6]. It has been shown that the PCR test is rapid, cheap, sensitive and specific test which can be used to replace the culture and RADT test used today for the diagnosis of GAS pharyngitis [7]. Although the methods of detection differ, all tests are based on GAS sampling using a throat swab of the tonsils and the posterior pharynx [5]. However, obtaining a throat swab is a complicated procedure for non-medical personnel, is often accompanied by fear and apprehension in children, and can elicit vomiting.

The use of saliva as a diagnostic specimen has recently attracted growing attention. It is easily collected and can be used as a target for the detection of different disease states. The use of saliva in the diagnosis of various bacterial infections such as periodontitis, mycoplasma respiratory infections and gonococcal pharyngitis has been described [8–10]. Thus saliva may be a promising way to detect GAS less invasively and can also be collected by parents. The aim of this study was to explore whether saliva sampling for the detection of GAS DNA followed by PCR amplification of specific GAS DNA sequences could constitute a viable non-invasive method for detecting GAS pharyngitis.

## Methods and materials

This prospective study was conducted at the Meuhedet Health Maintenance Organization clinic (HMO), Jerusalem, Israel, between 1 September 2017 and 1 August 2018. The study was approved by the ethics committee of the Hadassah Medical Center and the Meuhedet HMO. Informed consent was obtained from all subjects.

### Study population

Subjects were eligible if they had a clinical diagnosis of pharyngitis or tonsillitis based on clinical suspicion by the physician corresponding to the Centor scoring criteria, had no recent previous antibiotic therapy, no substantial comorbidities no chronic illnesses besides asthma, and no evidence of GAS carriage. GAS carriage was excluded based on clinical symptoms. Subjects were excluded if they had comorbidities or were already being treated with antibiotics. For each subject, a throat swab from the posterior oropharynx was obtained for culture and a saliva sample (up to 0.5 ml) was taken in a plastic container. The saliva sample was obtained by voluntary spitting into the container after the swab was obtained. The clinical data were documented for each patient, including the presence of fever (>38°C), additional symptoms, previous GAS pharyngitis infections, GAS pharyngitis in other family members and length of time since the first manifestations of the symptoms. In addition, relevant information from the physical exam such as tonsillar exudate and lymphadenopathy were documented.

### Throat culture

Swab specimens taken from patients were plated on blood agar and incubated overnight at 37**°**C in anaerobic conditions. Beta hemolytic colonies were taken for identification on VitekMS (BioMérieux, France).

### PCR testing

For purposes of control prior to testing, the system was calibrated by a spiking method where GAS colonies were added to GAS free saliva and the PCR parameters were defined. Then a PCR was conducted on 20 saliva samples for which the throat culture results were already available.

All saliva samples were stored at 4°C for 1–7 days prior to extraction of DNA. Bacterial DNA was extracted from the samples, and real time PCR (RT-PCR) was performed for the detection of the GAS *dnaseB* specific gene. The amount of saliva was measured and assigned to one of four groups as follows: + less than 30 μl, ++ 50–100 μl, ++ 100–150 μl, ++++ 200 μl and above. To extract the bacterial DNA, the sample volume was topped to 200 µl with sterile water, boiled at 100°C for 10 minutes, frozen at −20°C for 20 minutes and then centrifuged at 1000 rpm for 10 minutes. Amplification was performed by a Rotor-Gene Q (QIAGEN), with an initial cycle of 30 seconds at 95°C, followed by 40 cycles of 10 seconds at 95°C, 10 seconds at 60°C and 10 seconds at 72°C. The samples were tested for *dnaseB* with the Kapa SYBR® FAST qPCR Kit (KAPA Biosystems, Wilmington, Massachusetts) using a *dnaseB*-F and *dnaseB*-R primers mix (Slinger et al. 2011). The PCR mixture contained 2 µl of sample DNA within a total volume of 25 µl. [11]. All the samples were processed and the PCR was done by the same laboratory technician. Throat swabs were sent to the Meuhedet HMO Microbiology laboratory for bacterial culture.

### Statistical analysis

Results were analyzed using SPSS 21.0 (SPSS Inc., Chicago, IL) statistical software package. Comparisons of the throat cultures (gold standard) to the RT-PCR results were performed. To test for significant differences between groups, Chi-square and t- tests were conducted at a significance level of 0.05. All reported p-values are two-tailed. The data were analyzed for performance measures including sensitivity and specificity as well as positive and negative predictive values. Sample size was calculated with WinPepi. Assuming that 7–10% detection rate difference is considered negligible and expecting a total of 5–10% discrepant results, we would need 96–102 pairs of observation with a power of 80% and an alpha of 5% [12]

A Receiver operating characteristics (ROC) curve and area under the curve (AUC) were calculated.

## Results

A total of 102 subjects were enrolled. One subject dropped out of the study, and another subject was excluded due to loss of the saliva sample. A total of 100 subjects underwent analysis. The overall prevalence of GAS was 55% and 48% as determined by the throat culture and saliva PCR, respectively. The average age (±SD) of the patients was 8.4 years (±2.6); 51% were females. Twenty- five percent of the subjects had GAS pharyngitis in the previous six months and 17% had an additional family member with GAS pharyngitis. Of the children who had a GAS infection in the 6 months prior to the study, 58% had a positive culture. Seventy- two percent of the subjects had fever, 45% had exudates and 20% lymphadenopathy. Of the patients with a positive culture, 51% had exudates, 78% had fever and 18% had enlarged cervical lymph nodes. The average length of illness prior to medical assessment was 1.4 days (±0.7).

**Figure 1.**
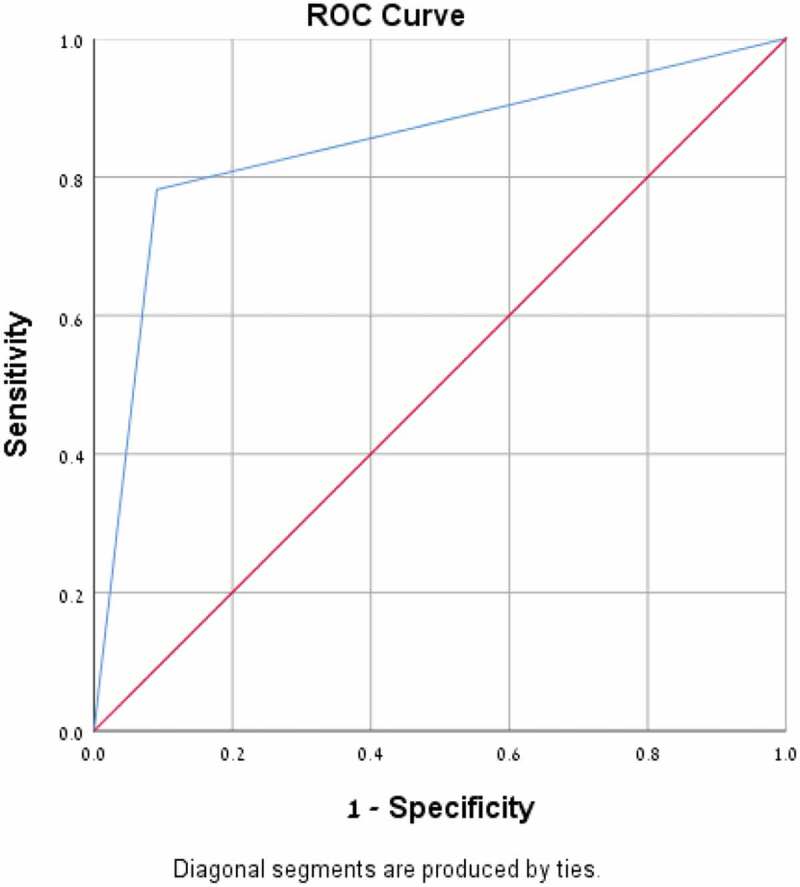
Receiver operating characteristics (ROC) Curve for saliva PCR for the diagnosis of GAS infection. Area under the curve (AUC) for saliva PCR was 0.85 (P < 0.001).

No statistically significant difference was found between the culture positive and culture negative cohorts for the above parameters apart from commencing empirical antibiotic treatment (p < 0.007) (Table 1). No statistically significant difference was found between the culture positive and the saliva PCR positive cohorts for the above parameters either (Table 2). Fifty-six throat cultures were positive for the presence of GAS and 44 were negative. Forty- eight saliva PCR samples were positive for the presence of GAS and 52 were negative. Twelve samples were throat culture positive but negative on the saliva PCR test and 4 samples were positive on the saliva PCR test but negative on the throat culture. The overall sensitivity of the saliva PCR test was 79%, with a specificity of 91%, PPV 92% and NPV 77% (Table 3). A ROC curve and AUC were calculated (Figure 1), AUC was found to be 0.85 (P < 0.001).

**Table 1.** Comparison of demographic and clinical characteristics: patients with positive and negative throat cultures.

	Positive culture N = 56	Negative culture N = 44	P Value
Age, years (SD)	8.6 (±3)	8.1(±2.2)	0.41
Female, n (%)	28 (50%)	21 (43.7%)	0.69
GAS in the past 6 months, n (%)	12 (21.4%)	13 (27.1%)	0.45
GAS in the family, n (%)	12 (21.4%)	5 (10.4%)	0.15
Days of disease, days (SD)	1.4(±0.6)	1.5(±0.7)	0.89
Exudate, n (%)	29 (51.8%)	16 (33.3%)	0.11
Fever, n (%)	43 (76.8%)	29 (60.4%)	0.22
Cervical lymph node enlargement, n (%)	10 (17.9%)	10 (20.8%)	0.62
Empirical ABX treatment, n (%)	35 (62.5%)	15 (31.3%)	0.007

**Table 2.** Comparison of demographic and clinical characteristics: patients with positive throat cultures and positive PCR for GAS.

	Positive culture N = 56	Positive GAS PCR N = 48	P Value
Age, years (SD)	8.6 (±3)	8.5 (±2.8)	0.89
Female (%)	28 (50%)	23 (47.9%)	0.78
GAS in the last 6 months (%)	12 (21.4%)	11 (22.9%)	0.91
GAS in the family (%)	12 (21.4%)	10 (20.8%)	0.85
Days of disease, days (SD)	1.4(±0.6)	1.4 (±0.6)	0.93
Exudate (%)	29 (51.8%)	22 (45.8%)	0.54
Fever (%)	43 (76.8%)	37 (77.1%)	0.97
Cervical lymph node enlargement (%)	10 (17.9%)	7 (14.6%)	0.65
Empirical ABX treatment (%)	35 (62.5%)	32 (66.7%)	0.66

**Table 3.** Comparison of sensitivity and specificity of Saliva PCR vs. Bacterial cultures as a function of the number of days since manifestation of symptoms.

	All days	Day 1	Day 2–4
sensitivity	79%	75%	90%
specificity	91%	85%	100%
PPV	92%	86%	100%
NPV	77%	73%	85%

An additional analysis of the saliva PCR method versus the throat culture revealed no correlation between the amount of saliva in the sample, the presence of tonsillar exudates, the presence of fever, age or the overall sensitivity and specificity of the method.

Samples taken on days 2–4 of the disease demonstrated a sensitivity of 90%, a specificity of 100%, a PPV of 100% and a NPV of 85%. By contrast, samples taken on the first day of the disease demonstrated a sensitivity of 75%, a specificity of 85%, a PPV of 92% and a NPV of 77% (Table 3). Twelve samples had a positive culture and a negative PCR. Eight of these samples (66%) were taken on day 1 of the disease, and two (16%) were taken on day 2, whereas two samples had no data on the day of illness. Four samples had a negative culture and a positive PCR, all of which were obtained on the first day of the illness.

## Discussion

Sore throat and tonsillitis are one of the most common complaints for which children seek medical advice. In order to confirm or rule out GAS infection of the pharynx or tonsils, a bacterial swab is obtained in selected cases from the posterior pharynx and tonsils followed by culture on appropriate media [1]. While only annoying and unpleasant in adults, the technique of obtaining a throat swab in children may cause considerable anxiety and apprehension. Furthermore, the results take 2–3 days when the cultured specimen is diagnostic [2,6]. Although numerous methods of GAS detection in suspected tonsillitis are available such as the traditional bacterial culture, the RADT and even PCR based kits [13,14] they all rely on sampling the posterior pharynx and tonsils with a swab.

By using a saliva – PCR based detection method, we aimed to find a test that would be fast and accurate, easily obtained by parents, and less conducive to the anxiety and apprehension children experienced with the throat swab technique. Saliva is more easily obtained and stored [15–17]. The use of saliva based PCR for the diagnosis of infectious diseases has been reported for periodontal diseases [10], Neisseria gonorrhoeae [8] and Mycoplasma pneumoniae infections [9].To the best of our knowledge, however, we are the first to report the use of this method in the diagnosis of tonsillitis. In one study comparing swabs taken from the oral cavity using different detection methods, PCR had a 40% sensitivity, RADT had a 20% sensitivity, whereas culture had an 80% sensitivity. All methods showed a specificity of 100%[18]. Another study using saliva- based PCR in periodontal disease reported a sensitivity of 86–89% and a specificity of 84–95% [19].

In the current study, the overall sensitivity and specificity were 79% and 91% respectively. This compares favorably to the 70–90% sensitivity and 95% specificity of the RADT test, but is lower than the 99% sensitivity and 99.6% specificity for the swab-based PCR test [2,4,6]. It is worth noting that when we subdivided our cohort into early (first day) and late (days 2–4) specimen collection, a significant improvement in the overall yield was obtained, in that the sensitivity on day 1 was 75% whereas on days 2–4 it improved to 90%. The specificity on day 1was 91% but rose to 100% on days 2–4. The difference in yield was apparent in both the false positive and the false negative measures. One possible explanation is that the inoculum of the bacteria in the posterior pharynx is still low on the first day of the illness and in some cases below the detection threshold of the saliva based PCR kit, hence producing false negatives. The false positive cases may have resulted from failure to touch an infected surface when obtaining the swab due to the low inoculum, whereas there were enough PCR fragments in the saliva to be detected by our method.

It could be argued that the saliva technique only obtained saliva from the proximal oral cavity. However, this explanation fails to account for the discrepancy between the early and late yield results.

### Limitations

This study has several limitations, the first of which was the small cohort. In addition, the saliva samples were stored in appropriate cooling conditions up to a week before PCR performance, whereas the swabs were immediately sent to culture. Furthermore, the saliva samples were taken after the pharyngeal swab, and this might have spread bacteria from the pharynx to the saliva, thereby enhancing the detection rate.

Nevertheless, this study is the first to report a PCR detection method for GAS using saliva samples. While this noninvasive method demonstrated overall good sensitivity and specificity for GAS detection, further studies are needed to optimize the yield and general performance of the method.

## References

[1] Shulman ST, Bisno AL, Clegg HW, et al. Clinical practice guideline for the diagnosis and management of group a streptococcal pharyngitis: update by the infectious diseases society of America. Clin Infect Dis. 2012;55(10):1279–1282. 10.1093/cid/cis847 23091044

[2] Steed LL, Korgenski EK, Daly JA. Rapid detection of streptococcus pyogenes in pediatric patient specimens by DNA probe. J Clin Microbiol. 1993;31(11):2996–5. 8263185 10.1128/jcm.31.11.2996-3000.1993 PMC266185

[3] Kimberlin D, Brady M, Jackson M, et al. Committee on infectious diseases. Group A streptococcal infections. Am Acad Pediatr. 2015; 732–744. Red Book.

[4] Tanz RR, Gerber MA, Kabat W, et al. Performance of a rapid antigen-detection test and throat culture in community pediatric offices: implications for management of pharyngitis. *Pediatrics*. 2009;123(2). DOI:10.1542/peds.2008-0488. 19171607

[5] Gerber MA, Shulman ST. Rapid diagnosis of pharyngitis caused by group a streptococci. Clin Microbiol Rev. 2004;17(3):571–580. 10.1128/CMR.17.3.571-580.2004 PMC452552 15258094

[6] Anderson NW, Buchan BW, Mayne D, et al. Multicenter clinical evaluation of the illumi gene group A streptococcus DNA amplification assay for detection of group A. J Clin Microbiol. 2013;51(5):1474–1477. 23447639 10.1128/JCM.00176-13 PMC3647941

[7] Cohen DM, Russo ME, Jaggi P, et al. Multicenter clinical evaluation of the novel alere i strep a isothermal nucleic acid amplification test. J Clin Microbiol. 2015;53(7):2258–2261. 25972418 10.1128/JCM.00490-15 PMC4473182

[8] Chow EPF, Tabrizi SN, Phillips S, et al. Neisseria gonorrhoeae bacterial DNA load in the pharynges and saliva of men who have sex with men. J Clin Microbiol. 2016;54(10):2485–2490. . 10.1128/JCM.01186-16 PMC5035428 27413195

[9] Komatsu H, Tsunoda T. Successful use of saliva without DNA extraction for detection of macrolide-resistant Mycoplasma pneumoniae DNA in children using LNA probe-based real-time PCR. J Infect Chemother. 2013;1087–1092. DOI:10.1007/s10156-013-0630-9. 23771735

[10] Zhou X, Liu X, Li J, et al. Real-time PCR quantification of six periodontal pathogens in saliva samples from healthy young adults. Clin Oral Investig. 2015;19(4):937–946. 10.1007/s00784-014-1316-0 25217278

[11] Slinger R, Goldfarb D, Rajakumar D, et al. Rapid PCR detection of group a streptococcus from flocked throat swabs: a retrospective clinical study. Ann Clin Microbiol Antimicrob. 2011;10(1):33. 21888649 10.1186/1476-0711-10-33 PMC3179694

[12] Abramson JH. WINPEPI updated: computer programs for epidemiologists, and their teaching potential. Epidemiol Perspect Innov. 2011;8(1):1. 21288353 10.1186/1742-5573-8-1 PMC3041648

[13] Felsenstein S, Faddoul D, Sposto R, et al. Molecular and clinical diagnosis of group A streptococcal pharyngitis in children. J Clin Microbiol. 2014;52(11):3884–3889. 25143573 10.1128/JCM.01489-14 PMC4313219

[14] Iverson LK, Santrach PJ, Henry NK, et al. Comparison of LightCycler PCR, rapid antigen immunoassay, and culture for detection of group A streptococci from throat swabs comparison of lightcycler PCR, rapid antigen immunoassay, and culture for detection of group A streptococci from throat swabs. J Clin Microbiol. 2003;41(1):242–249. 12517855 10.1128/JCM.41.1.242-249.2003 PMC149598

[15] Suzuki N, Yuyama M, Maeda S, et al. Genotypic identification of presumptive streptococcus pneumoniae by PCR using four genes highly specific for S. pneumoniae. J Med Microbiol. 2018;903(2006):709–714. 10.1099/jmm.0.46296-0 16687588

[16] Floriano PN, Christodoulides N, Miller CS, et al. Use of saliva-based nano-biochip tests for acute myocardial infarction at the point of care: A feasibility study. Clin Chem. 2009;55(8):1530–1538. 19556448 10.1373/clinchem.2008.117713 PMC7358990

[17] Nagler RM. Saliva analysis for monitoring dialysis and renal function. Clin Chem. 2008;54(9):1415–1417. 18755903 10.1373/clinchem.2008.112136

[18] Fox JW, Marcon MJ, Bonsu BK. Diagnosis of streptococcal pharyngitis by detection of Streptococcus pyogenes in posterior pharyngeal versus oral cavity specimens. J Clin Microbiol. 2006;44(7):2593–2594. 16825390 10.1128/JCM.00797-06 PMC1489465

[19] Saygun I, Nizam N, Keskiner I, et al. Salivary infectious agents and periodontal disease status. J Periodontal Res. 2011;1:235–239. 10.1111/j.1600-0765.2010.01335.x 21261620

